# The feasibility of an education video for preventing disease-related malnutrition among home-living older adults after discharge from a surgical hospital department — a randomised controlled feasibility trial

**DOI:** 10.1186/s40814-025-01745-y

**Published:** 2025-12-05

**Authors:** Monica Christin Hansen, Lisbeth Uhrenfeldt, Kari Ingstad, Preben U. Pedersen

**Affiliations:** 1 Faculty of Nursing and Health Sciences, Nord University, Universitetsalleen 11, Bodø, 8026 Norway; 2 Orthopaedic department, Kolding Hospital, Kolding, Denmark; 3 Department of Regional Health Science, Southern Danish University, Odense, Denmark; 4 Faculty of Nursing and Health Sciences, Nord University, Levanger, Norway; 5 Centre of Clinical Guidelines, Department of Clinical Medicine, Aalborg University, Aalborg, Denmark

**Keywords:** Feasibility study, Malnutrition, Disease-related malnutrition, Undernutrition, Education video, RCT, Knowledge translation, Nursing

## Abstract

**Background:**

Older adults can have limited knowledge about their specific needs for protein and energy and the importance of nutrition for physical functioning after hospital discharge. This study aimed to examine the feasibility of recruitment, technology access, and data collection procedures in an educational video aimed at improving the nutritional status of live-at-home older adults after discharge from a surgical hospital department.

**Methods:**

This seven-month single-centre, two-arm feasibility trial was conducted in a rural area in northern Norway from May 2022 to January 2023. The participants were live-at-home older adults 65 years of age or older, who were recruited from three surgical departments in a non-university hospital. The participants were randomised using a free web service. The intervention group received access to a six-minute nutrition education video focusing on energy and protein-rich diets for malnourished older adults. The control group did not receive any intervention. Data were collected on the study feasibility recruitment rate (goal: 5 people every 2 weeks over 28 weeks), retention rate (goal: 82%), and access to the video (goal: 90%) as well as the feasibility of—collecting data on the participants’ nutritional knowledge, body mass index, mid-arm circumference, triceps skinfold thickness, mid-arm muscle circumference, and hand grip strength.

**Results:**

Forty-four participants were randomised to the intervention group (*n* = 24) or the control group (*n* = 20). The recruitment rate was, on average, 3 patients every 2 weeks. The overall retention rate was 68%, respectively 71% (17), in the intervention group and 65% (13) in the control group. Collecting the desired data was feasible with specific muscle strength and fat mass adjustments. No adverse events related to participation in the study were observed.

**Conclusions:**

Delivery of the education video was feasible. However, the study revealed methodological challenges with recruitment and access to the video that must be addressed before a full-scale trial. The results indicate that nutrition education videos can be a good source of knowledge and contribute to a better nutritional situation for older malnourished adults. Still, it is necessary to conduct a full-scale trial in the future to conclude whether, or not, the nutrition education video’s is effective.

**Trial registration:**

This study was retrospectively registered in ClinicalTrials.gov ID NCT05860140.

**Supplementary Information:**

The online version contains supplementary material available at 10.1186/s40814-025-01745-y.

## Key messages regarding feasibility

- What uncertainties existed regarding the feasibility of this approach?
  Whether a nutritional educational video could be an effective intervention strategy to translate nutrition recommendations to prevent disease-related malnutrition among older adults remains to be determined. We conducted a randomised controlled feasibility study to investigate if it was feasible to recruit patients so they could view the video and if relevant outcome data could be collected in preparation for a future large-scale study.
- What are the key feasibility findings?
  Recruiting patients and eligible participants to watch the educational video was possible. However, the findings show that essential data were missing in patients’ records, eligible patients were missed, and patients had challenges viewing the video. It is possible to collect data regarding older adults’ nutrition knowledge and body composition measurements with some adaptations.
- What are the implications of the feasibility findings for the design of the main study?
  The findings indicate that it is feasible to carry out a randomised controlled study to assess the feasibility of recruitment, technology access, and data collection procedures in an educational video intervention aimed at improving the nutritional status of live-at-home older adults after discharge from a surgical hospital department. However, procedural changes must be made to address recruitment procedures and the feasibility of watching the educational video.

## Background

The prevalence of malnourished hospitalised older adults is estimated to be 20–50%, with further declines expected during hospitalisation [1, 2]. Further, it has been shown that about 33% of community-dwelling adults receiving home care are at risk for malnutrition [3]. A systematic literature review by Host et al. [4] found that age-related changes among older adults can lead to reduced intake of protein-rich foods and fruits/vegetables due to factors such as poor dental health, reduced appetite, and impaired sense of taste. Host et al. [4] further found that psychosocial aspects, such as loneliness, self-perceived health, quality of life, isolation, and low motivation to eat or cook alone, may influence older adults’ eating habits. At least personal resources, such as transportation options, finances, and nutrition knowledge, have been shown to correlate with the ability to maintain a healthy diet [4]. During hospitalisation, surgical stress can further exacerbate older adults’ pre-existing fluid and electrolyte imbalances [5]. Moreover, older adults often have a diminished capacity to compensate for fluid loss, blood loss, and electrolyte disturbances that arise in connection with illness and injury [5]. Thus, healthcare personnel need more time and nutrition-related training to provide nutritional education information and counselling to older adults [6]. Even if nutritionists can provide individual nutritional follow-ups to patients, it has been revealed that patients do not necessarily receive nutrition care plans or recommendations that could improve their outcomes [7]. As a result, older patients can have limited knowledge about their specific needs for protein and energy and the importance of nutrition for physical functioning [8]. Patients who lack access to such information after a hospital stay have reported difficulty in their transition from hospital care to self-management at home [9]. Earlier research has shown that older adults are often excluded from clinical trials due to logistical challenges, strict inclusion and exclusion criteria, and financial constraints [10]. In addition, advanced age, ill health, and difficulty understanding the study concept or randomisation may also affect older adults’ willingness to participate in a randomised controlled study. Consequently, older hospitalised patients with disease-related malnutrition may be a challenging group to include in a study [11]. In Pedersen, Pedersen, and Damsgaard’s [12] randomised clinical trial on nutritional follow-up after discharge from the hospital, 91 of 299 informed patients declined to participate. Earlier studies found that patients’ medical records can be incomplete or have missing data [13–15]. For example, a single-centre cross-sectional study found that only one of three medical patients had been screened for nutritional risk in a Danish University Hospital [16].

Different assessment tools can be used to detect nutritional challenges. Anthropological and body composition assessments are valuable tools for detecting nutritional challenges and effectively evaluating nutritional status during dietary interventions, including undernutrition and obesity [17]. In this context, anthropometry assessments offer an inexpensive, commonly applicable, portable, and noninvasive technique for assessing human body proportions, size, and composition [18]. In addition, screening tools such as the Nutrition Risk Screening Tool 2002, which assesses malnutrition or risk of malnutrition based on patients’ BMI, weight loss, appetite, and severity of illness, are commonly used in primary and specialist health care services [19].

Earlier studies on malnourished or at-risk individuals at discharge have focused on face-to-face contact and phone follow-up to determine nutritional status, which are resource-intensive procedures for an already strained healthcare system [20]. To reduce the risk of malnutrition, it is important to increase nutritional knowledge among older patients. However, to the best of our knowledge, there is a lack of studies addressing the efficacy of education videos in preventing disease-related malnutrition among older adults (aged above 65 years old) after a hospital stay.

A protocol created for a future full-scale intervention study served as a foundation for this study and has already been published elsewhere [21]. As this is the first intervention with the aim of changing patients’ nutritional behaviour using a video, an internal study was conducted to determine the feasibility of recruitment as well as collecting data on changes in nutritional behaviour and expected effect estimates on body composition. Feasibility studies are considered useful for estimating essential parameters needed to design a main study, such as participants’ willingness to be recruited and randomised estimations of effect sizes [22, 23].

## Methods

### Specific aim and research questions

This study aimed to examine the feasibility of recruitment, technology access, and data collection procedures in an educational video aimed at improving the nutritional status of live-at-home older adults after discharge from a surgical hospital department. Three research questions guided this study:

- 1: Is it feasible to recruit eligible participants in a full-scale randomised controlled trial that uses a digital educational nutritional video to transfer knowledge about nutritional intake?
- 2: Is it feasible to collect data regarding participants’ nutritional knowledge, body mass index (BMI), mid-arm circumference (MAC), triceps skinfold thickness (TSF), mid-arm muscle circumference (MAMC), and hand grip strength?
- 3: What are the challenges for live-at-home older adults in accessing a digital educational nutritional video?

### Trial design

We undertook a single-centre, two-arm, feasibility randomised controlled trial (RCT). This paper is reported in accordance with the extension of the Consolidated Standards of Reporting Trials (CONSORT) to pilot and feasibility trials [24].

### Research settings and participants

We conducted the study at a non-university hospital in a rural area in northern Norway between May 15, 2022, and January 6, 2023. The hospital trust serves about 136,000 people in 20 municipalities in the region. We recruited older adults (≥65 years) who received both surgical and nonsurgical treatment from one orthopaedic department. However, after 13 weeks, we also included two additional surgical departments to increase the inclusion rate. One was a urology and vascular/thoracic surgery department, and the other was a gastro, gynaecological, breast, and surgical endocrine department.

All potential participants that meet the following inclusion criteria were invited to participate: (a) home living with a home address in one of nine selected municipalities, which were chosen due to their physical proximity to the hospital and the research team location; (b) due to return home after discharge directly from the hospital or after a training stay at a rehabilitation centre before returning home; (c) able to read and understand Norwegian; (d) competence to provide consent; (e) access to a digital solution, such as a tablet or smartphone; (f) a BMI < 24 [25]. We planned to determine the potential participants’ BMI values through a nutrition screening in connection with their admission to the hospital. We excluded persons who were given only a liquid diet or were in the terminal phase.

Each department selected one to two recruiters with access to the department’s record system. The recruiters were registered nurses who received training from the first author regarding who could be assigned to participate in the study. The recruiters first screened the list of admitted patients 5 days per week according to the inclusion and exclusion criteria. Second, the first author physically visited the three hospital departments 5 days a week for 7 months to discuss with each recruiter which patients in the department would be suitable candidates for the study. The recruiters informed and obtained written consent from eligible patients. Both participants in the intervention group and the control group received a written information leaflet outlining the purpose of the study and the details of participation. The first author obtained the data material at the hospital and in the participants’ homes 3 months after discharge. The first author was also responsible for delivering the education video to the participants in the intervention group.

### Sample size

According to the CONSORT extension for feasibility and pilot trials statement, a formal sample size calculation is not required for feasibility and pilot trials [24]. However, recent guidance [26] recommends that sample size justification should be linked to the study’s primary feasibility outcomes. In this study, the main feasibility outcomes were the ability to recruit live-at-home older adults after hospital discharge, assess their access to digital technology, and evaluate the feasibility of data collection procedures. Based on the expected number of eligible patients discharged from the surgical department and previous experiences in similar populations, we anticipated recruiting approximately 70 participants over a seven-month period. This number was considered sufficient to estimate the recruitment rate and test the feasibility procedures with reasonable precision within the available time and resources. Due to limited resources, including time and funding, we opted to collect data over a span of 7 months and abstain from engaging in any ancillary analysis. Ethically, it is imperative that resources are utilised responsibly, with a priority placed on achieving the main study objectives. Since this was a low-risk intervention, there were no stopping rules or interim analysis.

### Intervention

#### Intervention group

The intervention group got access to a six-minute nutrition education video 5 days after discharge from the hospital. The nutrition educational video was accessible by clicking on a link sent out by the first author, and the participants accessed it via a link on their smartphone or email address. The first author developed the nutritional education video in consultation with the co-authors and a nutritionist. Two older adults who met the inclusion criteria but were not a part of the intervention also provided feedback on the language and content of the text. The video presented dietary recommendations from the Norwegian Diet Handbook (in Norwegian Kostholdshåndboken) of Helsedirektoratet [27]. The dietary guidelines recommend increasing protein and energy intake for older adults at risk for disease-related malnutrition or who have already been malnourished. Based on the Norwegian Diet Handbook, the video also gave examples of how older adults could adapt and integrate the recommendations into their daily mealtime routines. The participants were encouraged to watch the video several times.

#### Control group

The control group received only standard in-hospital care, meaning that no intervention was implemented for this group. However, based on the department’s standard routines, patients in both the intervention and control groups should undergo nutritional screening using the NRS2002, and a nutritionist would be consulted if necessary. Some patients also received home care that was unrelated to this study. Consequently, no concurrent treatment or intervention was taking place during the study.

### Randomisation and blinding procedure

After the participants gave their consent and the baseline data collection was completed, they were randomised based on their home municipality. We used the free software program RANDOM.ORG [28] to randomise the participants in block sizes of 10 to the intervention or control group (10:1). Due to the nature of this study, the participants became aware of whether they received access to the nutritional educational video, and thus it was impossible to achieve blinding. Furthermore, since the participants in the intervention group were going to be asked about the video’s availability at the follow-up 3 months later, it was not possible to achieve blinding when these data were collected.

### Statistical analyses

The feasibility outcomes regarding recruitment and access to the education video were reported narratively and descriptively. We used the software program SPSS Version 28.01 (IBM Corp., Armonk, NY, USA) for the statistical analysis. Baseline characteristics are presented for all ratio interval data, given as means, standard deviations (SDs), minimums, and maximums. Baseline characteristics based on nominal data are presented using valid N and percentages.

### Outcomes

The feasibility of the recruitment and intervention was assessed by measuring the recruitment rate, retention rate, and acceptance of the video.

#### Recruitment and retention rate

The recruitment rate was analysed by dividing the number of included participants by the number of weeks it took to recruit them. Success criteria for recruitment were defined as an average of 5 participants included every 2 weeks over 28 weeks [29–33].

The retention rate was defined as the percentage of participants enrolled at baseline who completed the follow-up measurements 3 months later. The criteria for retention rate success were estimated to be that at least 82.3% of participants completed the follow-up measurements 3 months later [29–33].

#### Acceptance of the intervention

The acceptance of the intervention was determined by measuring how many participants in the intervention group saw the video and the challenges that arose when the participants were offered the chance to watch the education video. Acceptance for the intervention was defined by a success rate of 90% [34]. Participants were further asked how many times they had seen the video, whether they had discussed the video with healthcare personnel, and if they had encountered any possible challenges in viewing the video at the follow-up 3 months after the intervention was delivered.

#### Nutrition knowledge

The participants’ nutritional knowledge was measured using a self-constructed questionnaire consisting of three questions where the participants were asked what they believed would be best to eat when they should start training and rehabilitation e.g. what they thought contained the most protein and energy of the following products: (1) fat or low-fat dairy products, (2) vegetables or meat products, and (3) apple or oat biscuit. The intervention group could find answers to the questions in the educational video. It is worth noting that the first author was present when the patients answered the questions.

#### Nutrition status

The participants were weighed wearing light clothes without shoes on an electronic Withings Body Cardio WBS04. Their weight was recorded to the nearest 0.1 kg, and their height was estimated based on their forearm length, counting the ulnar length between the point of the elbow and the midpoint of the prominent bone on the wrist. This value was compared with a standardised high conversion chart from RxKinetics [35] and was recorded to the nearest 0.5 cm. Participants’ BMI was then calculated as their weight (kg) divided by the square of their height (m).

Muscle strength was estimated by measuring hand grip strength using a Saehan Hydraulic Hand Dynometer and recorded to the nearest 0.5 kg for both the dominant and non-dominant hands. The participants were instructed to sit with their shoulder adducted and naturally rotated to the torso, the elbow flexed at 90 degrees, and the forearm and wrist in a neutral position [36]. The test was repeated three times on each arm, and the average value for each arm was recorded.

We planned to use the electronic Withings Body Cardio device to measure the participants’ muscle and fat mass. However, we eventually switched to manually measuring muscle and fat mass because the participants had challenges standing long enough to get the measurements registered on the device. Therefore, muscle mass was manually measured by estimating MAC using the midpoint between the olecranon and the acromion on the left arm, with the arm extended, muscles relaxed, and the palm facing the thigh [37]. The measurement was recorded in centimetres.

Finally, fat mass was manually estimated by measuring the TSF in mm using a VirtuFits Digital Fat Caliper on the triceps muscle on the participant’s left arm while the arm was hanging relaxed at the participant’s side. The test was repeated three times, and the average was recorded [38]. Based on the triceps skinfold thickness and midarm circumference, we calculated MAMC according to the following formula: MAMC = MAC – (3.14 × TFS thickness) [39].

## Results

### Recruitment and attrition

**Fig. 1:**
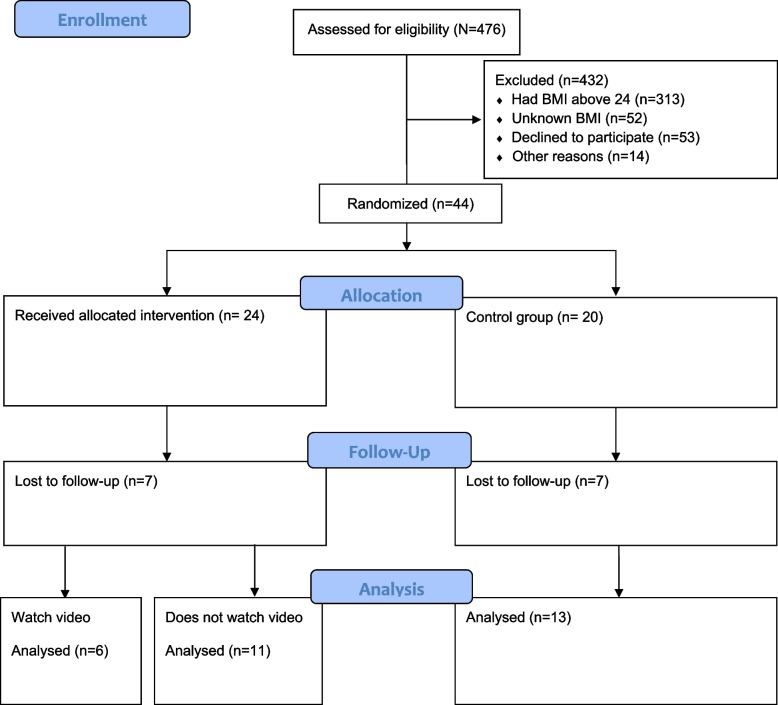
CONSORT flow diagram

A total of 476 patients in the three surgical departments met the eligibility criteria regarding age, municipal affiliation, capacity to consent, and satisfactory vision and hearing; see Fig. 1, the CONSORT FLOW diagram. These patients were not subject to restrictions like liquid food only or in the terminal phase.

When further screening for BMI, we found that nutrition screening had been done for 339 (71.2%) of these patients. Further, 85 (17.9%) patients had been enrolled since their height and weight were registered when they arrived at the hospital. However, 52 (10.0%) patients had no identified BMI and were excluded from participating.

After the first screenings, 111 patients were eligible for the study. Of these, 53 (48.0%) did not want to participate. These patients reported the following reasons for not participating in the study: lack of energy (*n* = 26); the project seemed too extensive (*n* = 6); physical challenges, such as impaired vision, pain, or nausea (*n* = 3); a perception that their digital competence was too low for participation (*n* = 3); they did not see a need for further knowledge about nutrition (*n* = 3); no desire to change their nutritional habits (*n* = 1); dissatisfaction with the treatment at the hospital (*n* = 1); and nine patients (*n* = 9) did not state any reason.

Of the 58 patients who were interested in participating further, 14 (24.0%) were excluded by the first author for the following reasons: cognitive impairment (*n* = 8); severely reduced physical condition (*n* = 3); liquid diet (*n* = 1); and severely reduced vision and hearing (*n* = 1).

Forty-four patients fulfilled the eligibility criteria, including having access to IT solutions, and they were randomised into two groups: one intervention group and one control group. Respectively, 24 participants were in the intervention group and 20 in the control group. The recruitment rate for the first 13 weeks, when participants were recruited from only one department, was on average 1 patient every 2 weeks (7 patients/13 weeks). When expanding to include two more departments, the recruitment rate increased to an average of 5 patients every 2 weeks (37 patients/15 weeks), giving a total recruitment rate on average of 3.2 patients every 2 weeks (44 patients/28 weeks), demonstrating the importance of increasing recruitment from several departments to achieve the goal of the recruitment rate.

Seven participants in the intervention group were lost to follow-up after 3 months, along with seven in the control group. Since the participants could withdraw their consent to participate in the study without justification, no information related to the reason for the dropout was collected. There were no statistically significant differences between participants who completed the study and those lost to follow-up, nor between the intervention and control groups.

A total of 30 participants completed the study, 17 in the intervention group and 13 in the control group. Six participants in the intervention group watched the education video, and for the per-protocol analysis, this group was called the video group.

### Number analysed

The demographic characteristics of the included participants and those lost to follow-up are given in Table 1. There was no difference between the participants in the inclusion and lost to follow-up groups. The sample demographic and clinical characteristics of all 30 participants who completed the study are provided in Table 2.

**Table 1:** Baseline characteristics of all the participants who agreed to participate in the study and were lost to follow-up

Patients’ characteristics	All participants (*N*=44)	Control (*n*=20)	Intervention (*n*=24)	Lost to follow-up (*n*=14)
**Age**, mean (SD) (min-max)	77,41 (7,70) (65–93)	76,60 (7,10) (65–92)	78,08 (8,23) (67–93)	77,14 (8,26) (65–93)
**Gender**, *n* (%)				
Female	28 (63,6)	9 (45,0)	19 (79,2)	10 (71,4)
Male	16 (36,4)	11 (55,0)	5 (20,8)	4 (28,6)
**Relative status, ***n* (%)				
Single	14 (31,8)	5 (25,0)	9 (37,5)	7 (50,0)
Partner/married	29 (65,9)	15 (75,0)	14 (58,3)	7 (50,0)
Other	1 (2,3)	0 (0,0)	1 (4,2)	0 (0,0)
**Education level, ***n* (%)				
Primary and secondary school	7 (15,9)	2 (10,0)	5 (20,8)	1 (7,1)
High school	26 (59,1)	11 (55,0)	15 (62,5)	11 (78,6)
College/university	11 (25,0)	7 (35,0)	4 (16,7)	2 (14,3)
**Receiving Home care, ***n* (%)				
Yes	7 (15,9)	2 (10,0)	5 (20,8)	3 (21,4)
No	37 (84,1)	18 (90,0)	19 (79,2)	11 (78,6)
**Discharge from the following surgical department, ***n* (%)				
Orthopaedic surgery	23 (52,3)	9 (45,0)	14 (58,3)	9 (64,3)
stomach/intestinal, breast, gynaecology and endocrine surgical diseases	9 (20,5)	6 (30,0)	3 (12,5)	4 (28,6)
Urology and vascular/thoracic surgery	12 (27,3)	5 (25,0)	7 (29,2)	1 (7,1)

**Table 2:** Baseline characteristics of all 30 participants who completed the study

Baseline characteristics	All participants (*n*=30)	Control (*n*=13)	Intervention (*n*=17)	Did not watch the video (*n*=11)	Did watch the education video (*n*=6)
**Age, **mean (SD) (Min-max)	77,53 (7,47) (65–92)	77,8 (7,61) (65–92)	77, 88 (7,75) (67–90)	76,5 (8,32) (67–90)	80,5 (6,19) (74–90)
**Gender, ***n* (%)					
Women	18 (60,0)	4 (30,8)	14 (82,4)	10 (90,9)	4 (66,7)
Men	12 (40,0)	9 (69,2)	3 (17,6)	1 (9,1)	2 (33,3)
**Relative status, ***n* (%)					
Single	7 (23,3)	1 (7,7)	6 (35,3)	3 (27,3)	3 (50,0)
Married/partner	22 (73,3)	12 (92,3)	10 (58,8)	7 (63,6)	3 (50,0)
Other	1 (3,3)	0	1 (5,9)	1 (9,1)	0
**Education level, ***n* (%)					
Primary and secondary school	6 (20,0)	2 (15,4)	4 ( 23,5)	3 (27,3)	1 (16,7)
High school	15 (50,0)	6 (46,2)	9 (52,9)	5 (45,5)	4 (66,7)
College/university	9 (30,0)	5 (38,5)	4 (23,5)	3 (27,3)	1 (16,7)
**Receiving Home care at baseline, ***n* (%)					
Yes	4 (13,3)	1 (7,7)	3 (17,6)	2 (18,2)	1 (16,7)
No	26 (86,7)	12 (92,3)	14 (82,4)	9 (81,8)	5 (83,3)
**Home care three months after inclusion, ***n* (%)					
Yes	7 (23,3)	4 (30,8)	3 (17,6)	3 (27,3)	0
No	23 (76,7)	9 (69,2)	14 (82,4)	8 (72,7)	6 (100,0)
**Hospital department, ***n* (%)					
Orthopaedic surgery	14 (47,75)	5 (38,5)	9 (52,9)	7 (63,6)	2 (33,3)
Stomach/intestinal, breast, gynaecology and endocrine surgery	5 (16,7)	4 (30,8)	1 (5,9)	1 (9,1)	0
Urology and vascular/thoracic surgery	11 (36,7)	4 (30,8)	7 (41,2)	3 (27,3)	4 (66,7)

### Feasibility of recruiting participants and access to the education video

Over a seven-month period, we recruited 44 patients who met all the inclusion criteria, including, for example, having access to the Internet. The total retention rate was 68%, respectively, and was similar between the two groups: 17 patients (71%) in the intervention group compared to 13 (65%) in the control group. We successfully sent the education video to all participants in the intervention group, while no patients in the control group received the video. Still, only six (35%) of the participants in the intervention group saw the education video. These participants each watched the video between one and four times, alone or with their relatives. None of the participants had discussed the content in the education video with healthcare personnel. The reasons the participants in the intervention group were unable to watch the education video were as follows: they did not remember receiving the video (*n* = 7); they forgot to watch the video because it was too much to handle after their hospital stay as e.g. strong pain and they were tired after operations (*n* = 3); they did not understand how to open the link to the video (*n* = 3); or they had not opened the link to the video because they were anxious about opening unknown links (*n* = 1). No adverse events related to the intervention were reported.

### The feasibility of collecting data regarding nutrition knowledge and body composition measurements

This feasibility study demonstrated that it was feasible to collect data on patients’ nutritional knowledge and body composition measurements. The questionnaire on nutrition knowledge was successfully filled out by all participants who finished all stages of the trial; however, not all body composition measurements were possible to perform on all of them. Since the participants had difficulty standing long enough to allow for a complete analysis using the electronic Withings Body Cardio WBS04, it was not feasible to collect data on body mass and body fat. We, therefore, converted to manually measuring fat and muscle mass, which was found to be a feasible approach. Measuring skinfold thickness was further challenging, as it could be difficult to distinguish between muscle tissue and fat. Still, weight, height, BMI, and handgrip strength were found to be feasible without modifications.

## Discussion

This study aimed to examine the feasibility of recruitment, technology access, and data collection procedures in an educational video aimed at improving the nutritional status of live-at-home older adults after discharge from a surgical hospital department. We demonstrated a recruitment rate of, on average, 3 patients every 2 weeks over a period of 7 months. In preparation for a future full-scale study, we utilised consecutive sampling in the data collection to provide insight into the number of eligible patients and the number of patients willing to participate in the intervention. There is no consensus in the literature regarding the appropriate sample size in feasibility/pilot studies, and the recommendations seem to vary from 10 to 12 per group to 60–75 per group, depending on the study’s primary objective [40]. Previous research has shown that it can be challenging to recruit older, frail adults for clinical studies [41, 42]. Thus, a strength of our study was that we were able to recruit older and potentially frail adults in a rural area for a digital nutrition intervention. Still, we were unable to recruit 5 participants every 2 weeks, which we could consider a success criterion for this feasibility study. Even if the success criteria are based on RCT studies from more populated areas in Denmark [29–33], a sample size means calculator [43] based on the results from our study found the necessary sample size for the main trial to be 138 participants for the intervention and control groups, respectively [21].

The following encountered challenges must be addressed before a future main study:

First, we must optimise the recruitment of participants by ensuring a well-implemented and established system for nutritional screening in the participating departments. This can be achieved, for example, through training on course days, close monitoring in the ward, and clear work divisions regarding the responsibility for performing nutritional screening.

Second, based on the feasibility study, it will be necessary to include more hospitals with associated surgical departments to recruit sufficient patients. We further strongly suggest that in a future full-scale study, we should have an even closer connection to the hospitals where the recruitment occurs, as only individuals working in the department have access to patients and patient records. Although the first author visited the hospital departments 5 days a week during the recruitment period to discuss potential participants who could be included in the study with the resource persons and motivate the resource persons to prioritise participation in the study, a possible solution may be for the recruitment personnel to work both in the hospital and at the university conducting the research. That could help to reduce the number of available personnel having to spend working hours on other pressing tasks, and we will not be dependent on resource persons in each department being at work and having the opportunity to screen possible participants to identify those who could be relevant for the study. The first author can then ensure that patients receive sufficient information about the study, which can help increase the recruitment rate and obtain consent from patients to participate in the study. Establishing a Patient and Public Involvement Panel (PPI Panel) comprising, for example, user representatives from patient organisations and nurses from the selected hospital can further be done to identify solutions that may enhance recruitment processes before the full-scale trial.

Thirdly, it must be facilitated to give participants more support, which ensures that participants follow the intervention by watching the video. Previous research has shown that older adults face challenges accessing and using digital solutions [44]. However, in our study, all the participants claimed they had access to internet solutions and the opportunity to watch the video. This bodes well for a future main study. No adverse effects of watching the education video were reported. At the same time, while the participants were encouraged to contact the research team in the event of any problems related to the education video, no one contacted the research team. We could not achieve a success rate of 90% of acceptance for the intervention. To increase the number of participants that follow the intervention, we suggest that the researchers help the participants with how to open the link to the video at the hospital, sending the participants a weekly text reminder with an invitation to watch the video or having a member of the research team call them after they have returned home from the hospital. Then, the participants could be reminded to open the video link and asked if they are experiencing any problems watching the video. Our feasibility study further indicates that patients who stated that they were in strong pain and were tired after operations ended up not watching the video, indicating that they lacked the motivation and energy to watch it. This shows that the patient had not necessarily received sufficient information about the purpose and length of the video, which we believe could have increased the likelihood that more participants would have watched the video. Additionally, it will therefore be relevant to invite a PPI panel to obtain input and discuss potential solutions that can enhance participants’ acceptance of the video in the future full-scale trial.

Fourthly, we must ensure that the data collector is well trained in using the calliper on older adults. The ageing process is characterised by a decrease in lean mass and bone density and an increase in total body fat mass, independent of general and physiological fluctuations in weight and BMI [45]. Measuring older adults’ skinfold thickness can be tricky since it can be difficult to distinguish between muscle tissue and fat [46, 47]. To strengthen the reliability of measuring the subcutaneous fat based on TSF, the first author performed all the measurements on each participant at baseline and 3 months after the participants were included in the study. Still, we experienced difficulty measuring the difference between adipose tissue and muscle with the calliper, which weakened the validity of this measurement tool in our study. Therefore, this will be addressed before the main trial.

Intervention techniques for transferring knowledge into practice include educational seminars, clinical guidelines, prompts, visual cues (e.g. symbols in the clinical area), audits, protocols, and leadership involvement [48, 49]. However, individual and organisational factors can be barriers to their implementation and uptake, including clinical behaviour, a lack of time and continuing education, and an unsupportive organisational culture [49, 50]. In our feasibility study, we wanted to test whether transferring knowledge through a digital education video could be feasible. We were therefore interested in whether the participants’ nutritional knowledge changed during the intervention. The participants’ nutritional knowledge was measured using questions the video group could answer based on the educational video. The first author was present with the participants when they answered the questions, ensuring they could not use a book or website to find the answers. However, to strengthen their validity, the questions about nutrition should have been validated before the feasibility study. The first author was available if the participants needed to discuss the meaning of the questions, and none of the participants expressed that they had problems understanding the questions.

Following Avery et al. [43] preliminary studies’ success in trial recruitment, protocol adherence, and completeness of outcome data may provide a guideline for assessing whether the full-scale study should be continued unchanged, continued with changes, or stopped before a full-scale trial. By making modifications to the protocol for the future full-scale trial, our study demonstrated that it can be feasible to enhance the success of study recruitment and the completeness of outcome data in the full-scale trial. Based on the results of the feasibility study, we argue that an educational video can be a potential tool for transferring knowledge regarding disease-related malnutrition and thereby can contribute to improving patient health outcomes, something that can contribute to improving the efficacy of the health system and reducing costs. At the same time, it is essential to mention that this study shows the analysis methods intended to be used in a future full-scale study where we have demonstrated that it was possible to collect data. Still, the data material was too small to conclude from the results. Future in-depth interviews of the participants could also reveal the participants’ experiences when watching the video, informing adjustments that will be necessary before the intervention is implemented on a larger scale.

### Limitations

One limitation of this study is that the person collecting data was also delivering the intervention, which means that the person collecting data was not blind to allocation. This is a major source of bias that, in a future full-scale trial, will be addressed by having different people in the research team be responsible for delivering the intervention as well as collecting and analysing data; the data collector will thus be blinded to the allocation.

A further limitation of our study is that we lack information on why the included departments had difficulties carrying out nutrition screening. Such information could have identified direct causes that we could address and act on. Still, it is well known that general barriers to early nutritional screening include insufficient knowledge by healthcare personnel regarding the role of nutrition screening and a lack of equipment and human resources [51]. Meanwhile, we know that good documentation systems for nutritional screening, personal motivation, and commitment on the part of hospital management seem to contribute to nurses’ focus on nutritional screening [52].

It has been discussions about whether the risk of disease-related malnutrition should be defined by either reduced BMI < 22 kg/m^2^ if >70 years, or non-volitional weight loss (>10% of habitual weight indefinite of time, >5% over 3 months, or >15 kg in the past 6 months) or reduced muscle mass (skeletal muscle index < 6.75 kg/m^2^ and 10.75 kg/m^2^ in females and males, respectively). Thus, a limitation may be that the inclusion criteria for participation in this study should have been set as a BMI < 22 kg/m^2^. However, we chose to include participants with a BMI < 24 kg/m^2^ since ill patients are in need of extra protein and energy to increase their survival and reduce the risk of complications during and after discharge from the hospital [25, 53]. As the video contained recommendations about what older malnourished people should consume in connection with illness, they were considered to benefit from the intervention regardless of whether their BMI was below 24 kg/m^2^ or 22 kg/m^2^. Simultaneously, it was therefore not desirable to offer the video to obese patients with unintentional weight loss, as they may need to integrate a restriction in calories with high protein administration to prevent loss of skeletal muscle mass and function [54–56].

In studies with a small number of participants, it can be challenging for the control group to serve as a true comparator. However, in our trial, both groups received identical standard care, including routine nutritional screening and access to nutritional consultations, ensuring that the control group represented a valid comparator. The intervention group additionally received the educational video, which is the component being tested. This design enables us to specifically evaluate the additional effect of the intervention beyond usual care in a future full-scale trial.

The small sample size increases the risk of skewed group characteristics. Since this was a feasibility study, the primary objective was to test the feasibility of recruitment, technology access, and data collection procedures of an educational video aimed at improving the nutritional status of live-at-home older adults after discharge from a surgical hospital department. Issues related to skewed group characteristics will therefore be addressed in the main trial, which will have a larger sample size and, consequently, a greater ability to detect meaningful differences.

At least, the nutrition knowledge questionnaire was self-constructed and not validated; its reliability is therefore uncertain. However, this was done so that the questions would be as closely aligned as possible with the knowledge the participants could acquire by watching the educational video.

## Conclusions

This study examined the feasibility of an educational video designed to improve the nutritional status of live-at-home older adults after being discharged from the hospital. The study was conducted in a Norwegian non-university hospital and included 44 participants. The results show that an educational video has the potential to be used as a tool to transfer knowledge to home-living older adults, helping to improve their nutritional status when they have recently been discharged from the hospital. By testing the study protocol for a full-scale trial in this feasibility study, we identified methodological challenges related to participant recruitment and measuring the desired outcomes. Adaptations related to recruitment and measurement instruments will thus strengthen the protocol for our future full-scale intervention. Moreover, the findings showed that only six participants watched the video, showing the need to make arrangements to ensure that the participants could watch the educational video. Overall, the findings of this study suggest that conducting follow-up studies, particularly through individual interviews with participants, would be valuable for gaining a deeper understanding of their experiences before the implementation of a full-scale intervention.

## Supplementary Information

Additional file 1: CONSORT checklist.

## Data Availability

All investigators involved in the study will have full access to the data. The datasets used are available from the corresponding author upon reasonable request.

## Abbreviations

BMI: Body mass index
CONSORT: Consolidated standards of reporting trials
MAC: The mid-arm circumference
MAMC: The mid-arm muscle circumference
PPI Panel: Patient and public involvement panel
SD: Standard deviation
RCT: Randomised controlled trial
TSF: Triceps skinfold thickness
